# The apid cuckoo bees of the Cape Verde Islands (Hymenoptera, Apidae)

**DOI:** 10.3897/zookeys.218.3683

**Published:** 2012-08-30

**Authors:** Jakub Straka, Michael S. Engel

**Affiliations:** 1 Department of Zoology, Charles University in Prague, Viničná 7, CZ-128 44 Praha 2, Czech Republic; 2 Division of Entomology, Natural History Museum, and Department of Ecology & Evolutionary Biology, 1501 Crestline Drive – Suite 140, University of Kansas, Lawrence, Kansas 66045, USA

**Keywords:** Apoidea, Anthophila, Apidae, cleptoparasite, taxonomy, *Thyreus*, *Chiasmognathus*, Cape Verde Islands

## Abstract

The apid cuckoo bees of the Cape Verde Islands (Republic of Cape Verde) are reviewed and five species recognized, representing two genera. The ammobatine genus *Chiasmognathus* Engel (Nomadinae: Ammobatini), a specialized lineage of cleptoparasites of nomioidine bees is recorded for the first time. *Chiasmognathus batelkai* **sp. n.** is distinguished from mainland African and Asian species. The genus *Thyreus* Panzer (Apinae: Melectini) is represented by four species – *Thyreus denolii* **sp. n.**, *Thyreus batelkai* **sp. n.**, *Thyreus schwarzi* **sp. n.**, and *Thyreus aistleitneri* **sp. n.** Previous records of *Thyreus scutellaris* (Fabricius) from the islands were based on misidentifications.

## Introduction

Although the bees of isolated archipelagos are of considerable interest and have usually attracted some degree of melittological interest, those species of the Republic of Cape Verde have been only anecdotally treated (Kirby 1884; Groh 1982; Simon Thomas and Wiering 1993; Báez et al. 2005), except for a contribution regarding samples of the family Halictidae (Pauly et al. 2002). There have been only 10 scientific expeditions which studied Hymenoptera in this archipelago over the course of 150 years of historical research (Table 1). Recent collecting efforts in the islands are therefore of considerable interest and provide greater information on the fauna. Herein we provide a taxonomic overview of the apid cuckoo bee fauna from the Cape Verde Islands, based on newly collected material as well as revision of material collected in the past. In total two genera are recognized. The genus *Thyreus* Panzer is represented by four species, all new and endemic to the islands, although some were misidentified as the otherwise more widespread Mediterranean and Asiatic species, *Thyreus scutellaris* (Fabricius) [(e.g., Kirby 1884; Báez et al. 2005 (both as “*scutellatus*”)]. Simon Thomas and Wiering (1993) suggested this was a mistake and correctly associated their material of *Thyreus* as related to *Thyreus ramosus* (Lepeletier de Saint Fargeau) (as “aff. *ramosus*”). The genus *Chiasmognathus* Engel is newly recorded from the Cape Verde Islands and is represented by a single new species, cleptoparasitic upon *Ceylalictus capverdensis* Pesenko, Pauly, and La Roche (Pauly et al. 2002). The only other parasitic bee known from the Cape Verde Islands is *Sphecodes pinguiculus* Pérez, formerly described as an endemic species (*Sphecodes capverdensis* Pauly and La Roche: Pauly et al. 2002) but recently recognized as this widespread Palearctic species and synonymized (Bogusch and Straka 2012). The host of *Chiasmognathus* was treated by Pauly et al. (2002), while species of the genus *Amegilla* Friese, the hosts of the *Thyreus*, are currently under study (Engel and Straka in prep.).

**Table 1.** A list of expeditions and names of collectors of bees who visited the Cape Verde Islands and provided their material for scientific studies.

**Collector(s)/Expedition(s)**	**Dates**	**Current Repository of Material**
HMS Challenger Expedition	1873	presumably NHML
L. Fea	1898	presumably MSNG, SEMC
H. Lindberg	1953–1954	MZH
A. van Harten	1963–1990	RMNH
E. Bauber, B. Friebe, K. Groh, H. Hölzel, W. Lobin, P. Ohm, B. Traub	1978–1980	FISC
R.T. Simon Thomas	1988	RMNH
F. La Roche	1998–1999	Personal coll., MICN
E. Aistleitner	1999–2009	Personal coll., NMPC, SEMC
J. Straka, J. Batelka	2009–2011	Personal coll., NMPC, SEMC

## Material and methods

The material considered herein is located in the following institutional and personal repositories: **EAFC**, Eyjolf Aistleitner, Feldkirch, Austria; **NMPC**, Department of Entomology, National Museum, Prague, Czech Republic; **FISC**, Forschungsinstitut Senckenberg, Frankfurt am Main, Germany, **JSPC**, Jakub Straka Collection, Charles University in Prague, Prague, Czech Republic; and **SEMC**, Division of Entomology, University of Kansas Natural History Museum, Lawrence, Kansas, USA. Abbreviations of additional repositories referred to in Table 1 are as follows: **NHML**, The Natural History Museum, London, United Kingdom; **MSNG**, Museo Civico di Storia Naturale “Giacomo Doria”, Genoa, Italy; **MICN**, Museo Insular de Ciencias Naturales, Tenerife, Canary Islands, Spain; **MZH**, Finnish Museum of Natural History, Helsinki, Finland; **RMNH**, Nationaal Natuurhistorische Museum (“Naturalis”), Leiden, The Netherlands.

Morphological terminology for the descriptions follows that of Engel (2001) and Michener (2007) except we utilize the annotations developed by Lieftinck (1962, 1968) for patches of plumose white setae on the mesosoma (outlined below), while the format for the descriptions follow those used elsewhere for Melectini and Ammobatini, respectively (e.g., Lieftinck 1968; Rightmyer and Engel 2003; Engel 2009, 2010). Although Lieftinck’s abbreviations for patches may not be intuitive, we believe it is imperative to maintain continuity between this work and his monumental effort to revise a difficult group of bees. However, in order to avoid confusion when using the descriptions and keys we have also inserted full names of these patches in parentheticals. Lieftinck’s abbreviations are:

als anterolateral mesoscutal patch (paired); frequently transverse in shape and along anterior margin and often contiguous with *lpn*.

deps dorsal mesepisternal patch; large patch covering upper portion of mesepisternum.

hypm hypoepimeral area patch; patch situated under wing base and posterior to pronotal lobe and often contiguous with *deps*.

lp lateral propodeal patch; conspicuous patch on either side of propodeum and often concealing spiracle.

lpn lateral pronotal patch (paired); transverse and placed on each side of middle on dorsal-facing surface of pronotum and toward posterior lobe.

mls mediolateral mesoscutal patch (paired); often round and situated on either side and slightly anterior to center of mesoscutum and just posterior to caudal apex of *ms*.

ms median mesoscutal patch (unpaired); anterior longitudinal patch from anterior of mesoscutum and extending caudad beyond *als* along median line; often relatively diffuse.

pls posterolateral mesoscutal patch (paired); round or hook-shaped patch in line with *mls*, sometimes projecting anteriorly along lateral border to meet *plsa*.

plsa anteriorposterolateral mesoscutal patch (paired); narrow, longitudinal patch along lateral border with tegula, often extending posteriorly to meet anteriolateral projection of *pls*.

ps parascutellar patch (paired), situated within the axilla.

s mesoscutellar patch (paired), typically just posterior to apicolateral angle.

t tegular patch, often posteriorly or along inner posterior angle.

veps ventral mesepisternal patch; smaller patch on ventral margin of mesepisternum.

**Map 1.**
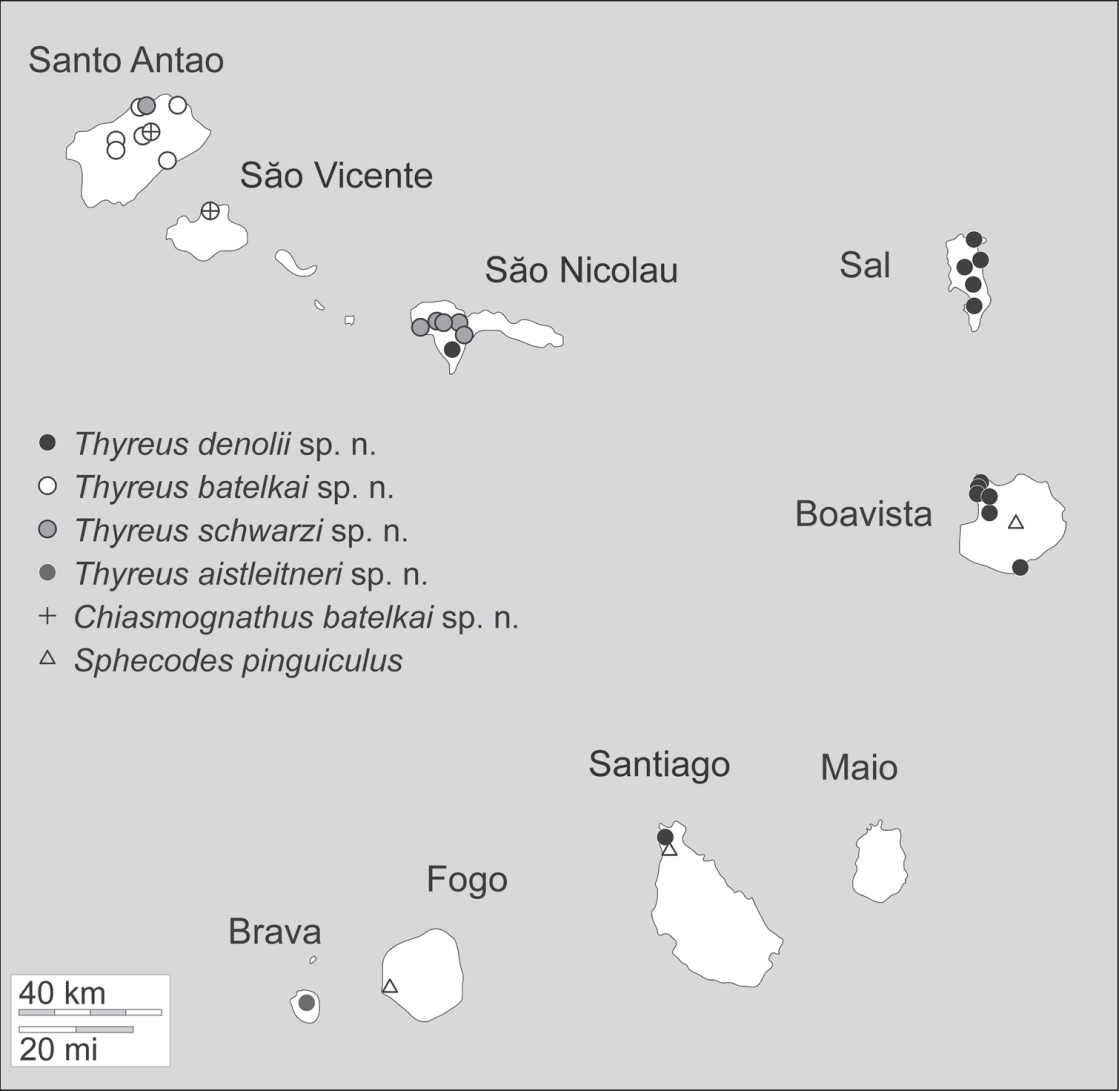
Cape Verde islands with distribution of all cuckoo bee species.

Photomicrographs were prepared using a Canon EOS 7D digital camera attached to an Infinity K-2 long-distance microscope lens. Measurements were made using an ocular micrometer attached to an Olympus SZX-12 or MBS-10 stereomicroscope. Distributions of the species across the various islands are summarized in Table 2 and Map 1 (outline map of Cape Verde came from d-maps.com).

**Table 2.** Currently confirmed distributions of cuckoo bee species across the Cape Verde Islands (see also Map 1). All of the apid species are endemic, while *Sphecodes pinguiculus* Pérez is widespread (Bogusch and Straka 2012). The distribution of *Sphecodes pinguiculus* in the Cape Verde islands is specified here for the first time.

**Taxon**	**Barlavento**						**Sotovento**			
	**Santo Antão**	**São Vicente**	**Santa Luzia**	**São Nicolau**	**Sal**	**Boa Vista**	**Maio**	**Santiago**	**Fogo**	**Brava**
*Chiasmognathus batelkai*	X	X								
*Thyreus denolii*				X?	X	X		X		
*Thyreus batelkai*	X	X								
*Thyreus schwarzi*	X?			X						
*Thyreus aistleitneri*										X
*Sphecodes pinguiculus*						X		X	X	
**Totals**	**3**	**2**		**2**	**1**	**2**		**2**	**1**	**1**

## Systematics

### Subfamily Apinae Latreille. Tribe Melectini Westwood. Genus *Thyreus* Panzer

The genus *Thyreus* is the most diverse lineage of Melectini, encompassing at least 104 previously described species (e.g., Meyer 1921, 1922; Lieftinck 1958, 1959a, 1959b, 1962, 1968; Rozen 1969; Eardley 1991; Schwarz 1993). It is also one of the more challenging genera in the tribe, with considerable variation within species, many closely similar (perhaps even cryptic) species, and little in the way of comprehensive biological data. This difficulty in characterizing species based on unique and fixed traits is highlighted by the existing monographs which have attempted to clarify the taxonomy of this lineage. While assuredly monophyletic, no consistent concept of intergeneric relationships has been established, although the *scutellaris* group of species (excluding those species transferred to the genus *Thyreomelecta* Rightmyer and Engel, 2003) are likely relatively basal in the genus as their mesoscutellar structure appears more primitive relative to that of all other *Thyreus*. Where known, *Thyreus* principally are cleptoparasitic on *Amegilla* Friese, although a few records do exist of certain species victimizing *Anthophora* Latreille (subgenera *Heliophila* Klug, *Mystacanthophora* Brooks, and *Dasymegilla* Brooks) and *Eucera* Scopoli (subgenus *Synhalonia* Patton). The Cape Verde Islands *Thyreus* parasitize *Amegilla* (*atrocincta* species group; = *Micramegilla* Brooks), commonly found in association in the field (pers. obs.).

#### Thyreus denolii sp. n.

urn:lsid:zoobank.org:act:6436F526-A122-4B3F-B173-74EF3DF4948F

http://species-id.net/wiki/Thyreus_denolii

**Figures 1–2.**
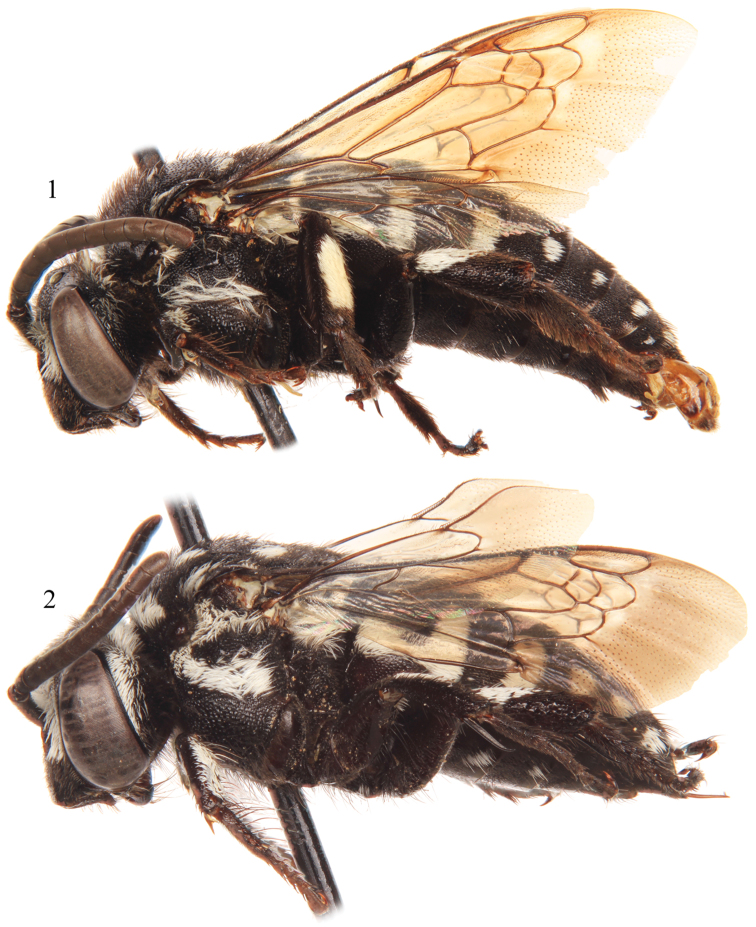
Lateral habitus of *Thyreus denolii* sp. n. from Boavista. **1** Male **2** Female.

Figs 1 [Fig F3] [Fig F4] –11

##### Holotype.

♂, Cape Verde Isl., Boavista, rock N of Sal Rei, 20.x.2009 [20 October 2009], J. Batelka & J. Straka lgt. (SEMC).

##### Paratypes.

**Boavista:** 1♂, 2♀♀, Cabo Verde 00/41, Ilha de Boavista, Sal Rei–S, 10 m, 30.12.2000 [30 December 2000], leg. Aistleitner (EAFC); 4♂♂, Cabo Verde, Boavista, Costa de BoaEsperança, (NE Sal Rei), 50 m, 1.1.2001 [1 January 2001], leg. Aistleitner/ 46 (EAFC); 2♀♀, Cabo Verde 00/47, Ilha de Boavista, Ribeira de Rabil, 10–20 m, 2.1.2001 [2 January 2001], leg. Aistleitner (EAFC); 3♀♀, Cabo Verde 00/48, Ilha de Boavista centr., Estancia de Baixo, 60 m, 2.1.2001 [2 January 2001], leg. Aistleitner (EAFC); 1♂, 3♀♀, Cape Verde Isl., Boavista – Sal Rei, on the beach, 1.X.2009 [1 October 2009], J. Straka & J. Batelka lgt. (JSPC, FISC); 1♂, 1♀, Cape Verde Isl., Boavista – Sal Rei, dunes, sweeping, 19.X.2009 [19 October 2009], J. Batelka & J. Straka lgt. (JSPC); 3♂♂, Cape Verde Isl., Boavista – rock N of Sal Rei, 20.X.2009 [20 October 2009], J. Batelka & J. Straka lgt. (JSPC); 1♀, same data as holotype except: Sal Rei on the beach, 1.x.2009 [1 October 2009], J. Straka & J. Batelka lgt. (SEMC); **Sal:** 1♂, 4.11.1980 [4 Noveber 1980], SAL, Straße Flughafen – Sta. Maria, n. v. Algodoeiro, Islas do Cabo Verde – 1980, H. Hölzel, W. Lobin, P. Ohm [collectors] (FISC); 1♂, 2♀♀, Cabo Verde 00/2, Ilha do Sal, Espargos, Boa Terra, 60 m, 28.11.2000 [28 November 2000], leg. Aistleitner (EAFC); 4♂♂, 3♀♀, Cabo Verde 00/6, Ilha do Sal, Pedra Lume, Ostküste, 20–40 m, 29.11.2000 [29 November 2000], leg. Aistleitner (EAFC); 1♂, Cabo Verde 00/7, Ilha do Sal, Mte. Grande–S, 70–170 m, 30.11.2000 [30 November 2000], leg. Aistleitner (EAFC); 1♂, the same as previous except 250 m (EAFC); 2♂♂, Cabo Verde 00/54, Ilha do Sal, Mte. Grande–E, 60 m, 9.1.2001 [9 January 2001], leg. Aistleitner (EAFC); 1♀, Cabo Verde, Ilha do Sal, Pedra Lume, 19.3.2004 [19 March 2004], leg. Aistleitner (EAFC); 1♀, Cape Verde Isl., Sal – Murdeira, ribeira, 2.–3.XI.2011 [2–3 November 2011], 16°41'N, 22°55'W, J. Batelka & J. Straka lgt. (JSPC).

##### Additional material.

**Sal:** 1♀, Cape Verde Isl., Sal – Monte Grande, 16.XI.2011 [16 November 2011], 20–350 m, 16°49'22"N, 22°54'22"W, J. Batelka & J. Straka lgt. [remnants of meso and metasoma] (JSPC); **Santiago:** 4♀♀; Ilhas do Cabo Verde, S. Tiago, Tarrafal, Triebe, Goh leg., 18.–20.10.79 [18–20 October 1979] (FISC, JSPC); 1♀, the same as previous except: Goh, Lobin leg., 10.79 [October 1979] (FISC); **São Nicolau:** 1♀, Ilhas do Cabo Verde, S. Nicolau, Goh, Lobin leg., 10.79 [October 1979] (FISC).

##### Diagnosis.

**Figures 3–4. F3:**
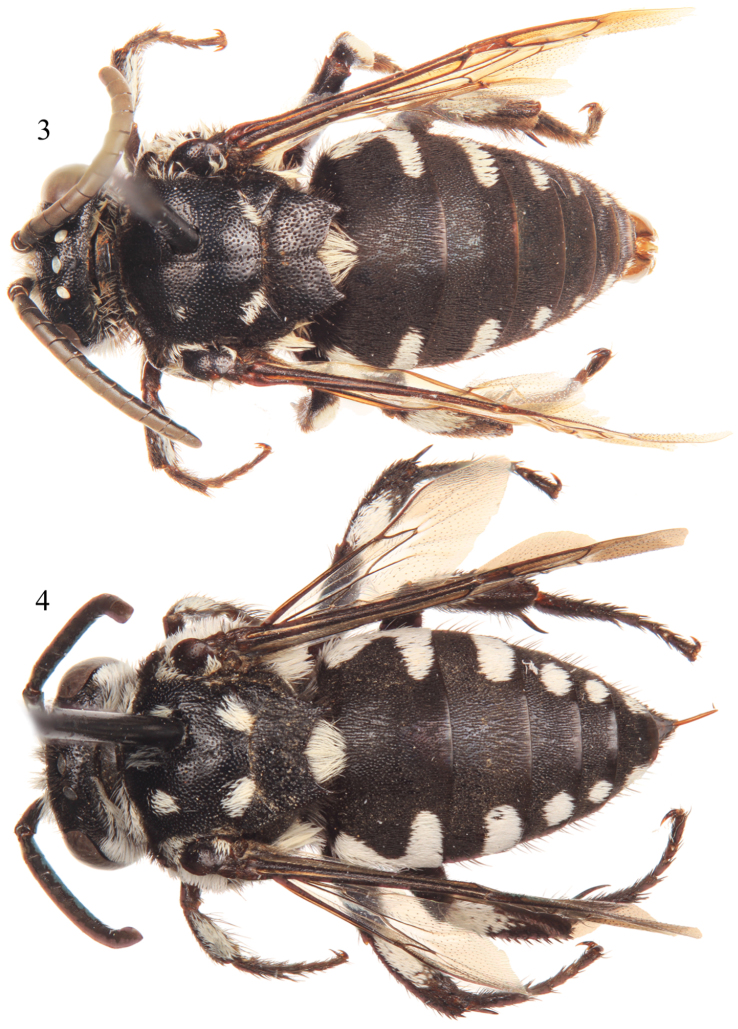
Dorsal habitus of *Thyreus denolii* sp. n. from Boavista. **3** Male **4** Female.

*Thyreus denolii* is one of the most distinctive of the Cape Verde Island species of *Thyreus*. The species can be readily recognized in the male by the sixth metasomal tergum with lateral white patches of the same size as on the fourth and fifth terga (rarely with the white patches of the sixth tergum reduced, but when so, then the patches are also reduced on the preceding terga) (Figs 1, 3). The female has a combination of the ventral and ventrolateral parts of the mesepisternum with distinct shiny interspaces among the punctures; the mesoscutum with *plsa* (anterior posterolateral mesoscutal) present and bordering the anterior portion of the tegula, but not meeting *pls* (posterolateral mesoscutal) posteriorly (Fig. 4); the apical depression of the fifth metasomal tergum densely punctate medially and densely setose; and the fifth tergum with lateral white patches of the same size as those on the fourth and third terga (Figs 2, 4). Both sexes have the combination of the apicolateral corners of mesoscutellum weakly pointed, forming an angle of more than 40° (although more sharply pointed in the female than the male), and the mesoscutellum finely punctate, with punctures separated, at least on the disc, by 0.5–1 times a puncture width.

##### Description.

♂: Total body length 9.9 mm (7.5–9.9 mm); forewing length 7.4 mm (6.0–7.9 mm). Head wider than long (length 2.4 mm, width 3.0 mm); upper interorbital distance 1.8 mm; lower interorbital distance 1.3 mm. Intertegular distance 2.4 mm (2.0–2.6 mm); mesoscutellar posterior margin often sinuate, sometimes weakly so, such that apicolateral angle projects as a prominent, broad spine and with a defined median emargination [degree of this sinuation is variable and so some males have spines and median emargination less prominent, but margins from apicolateral corners to midpoint are never straight as is usual for species such as *Thyreus hohmanni* Schwarz, typical *Thyreus ramosus* (Lepeletier de Saint Fargeau), or several Asiatic species]. Inner anterior angle of metatibia not swollen or projecting into prominence or point between metatibial spurs (e.g., in some Palearctic species this area of metatibia is prominently developed: e.g., Lieftinck 1968). Apex of seventh metasomal tergum with apicolateral prominences distinct, truncate margin between straight, without medial emargination or swelling; male terminalia as in Figures 7–11.

Labrum with coarse, irregular, contiguous punctures except basolateral impunctate areas, such areas longer than wide and therefore ovoid in shape, basomedially with short V-shaped furrow; clypeus with small contiguous punctures, integument between (where evident) smooth; face as on clypeus except punctures slightly larger, nearly contiguous, and somewhat weaker on supraclypeal area; punctures weaker and shallower on vertex and on ocellocular area, with small impunctate area bordering lateral ocellus; punctures coarse, shallow, and nearly contiguous on gena and posterior area of postgena, anterior area of postgena smooth and impunctate. Pronotum with coarse, shallow, nearly contiguous punctures; mesoscutum with well-defined punctures separated by less than a puncture width, slightly more widely spaced around parasidal lines and medioposteriorly such that punctures are separated by about 0.5–2 times a puncture width, integument between punctures smooth and shining; mesoscutellum, including axilla, with punctures as on medioposterior section of mesoscutum except punctures separated by 0.5–1 times a puncture width; pleura with coarse, nearly contiguous punctures, integument between punctures (where evident) smooth and shining; hypoepimeral area with impunctate area bordering scrobe; propodeal lateral and posterior surfaces with coarse, shallow, ill-defined, nearly contiguous punctures. Metasoma with small punctures separated by less than a puncture width, punctures coarser, larger, and somewhat more poorly defined on more apical terga, integument between finely imbricate, apical margins narrowly impunctate and finely imbricate; sterna with similar punctation except those on discs of more basal sterna more widely spaced and becoming more poorly defined on more apical sterna.

Integument black except dark brown on tarsi, mouthparts, and apically on seventh metasomal tergum and on apical sterna. Wing membranes hyaline and slightly infumate, veins dark brown to black.

**Figures 5–6. F4:**
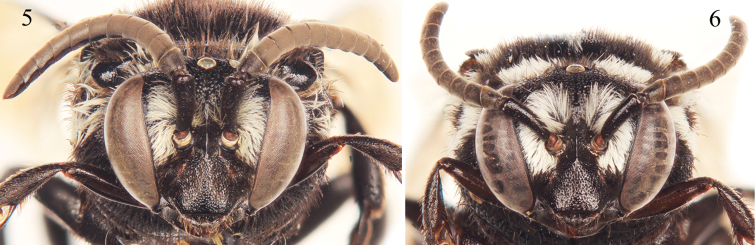
Facial aspect of *Thyreus denolii* sp. n. from Boavista (facial views for the various species do not really vary and so only those for *Thyreus denolii* are presented). **5** Male **6** Female.

Pubescence generally fuscous to black over entire body except for presence of plumose white setae on face (Fig. 5), posterior of vertex, upper gena, outer surface of protibia, outer surface of mesotibia, outer basal surface of metatibia, and on mesosoma (using annotation system of Lieftinck 1962, 1968) as follows: *deps* (dorsal mesepisternal) and *lpn* (lateral pronotal) present; *als* (anterolateral mesoscutal) present but reduced, often faint; *ms* (median mesoscutal) present, although often reduced and faint; *mls* (mediolateral mesoscutal) present; *plsa* (anterior posterolateral mesoscutal) present along anterior half to two-thirds of border with tegula, not meeting *pls* (posterolateral mesoscutal) posteriorly; *t* (tegular) present and prominent posteriorly on tegula; *pls* (posterolateral mesoscutal) present, not extending laterally to meet *plsa* (anterior posterolateral mesoscutal); *ps* (parascutellar) and *s* (mesoscutellar) absent; *deps* (dorsal mespisternal), *hypm* (hypoepimeral area), and *lp* (lateral propodeal) present, *veps* (ventral mesepisternal) absent (Figs 1, 3). Mesoscutellum with dense patch of long, plumose, white setae extending posteriorly from undersurface of mesoscutellum medially, patch wide but not reaching to apicolateral corners. Metasomal terga with prominent patches of appressed, plumose white setae as follows: first metasomal tergum with large, L-shaped patches laterally; second metasomal tergum with lateral patch transverse, slightly wider than twice as long, never L-shaped and without rounded secondary anterior patch; third through sixth metasomal terga with more or less transverse to rounded lateral patches (Figs 1, 3).

♀: As described for the male except in usual gender differences and as follows: Total body length 8.1–10.6 mm; forewing length 6.4–7.4 mm. Head wider than long (length 2.4 mm, width 3.0 mm); upper interorbital distance 1.9 mm; lower interorbital distance 1.3 mm (Fig. 6). Intertegular distance 2.1–2.5 mm; mesoscutellar posterior margin as in male but sometimes sinuate margin weaker and apicolateral angle not forming as prominent a spine. Apical depression of fifth tergum densely punctate and setose. Pygidial plate relatively narrow, margins converging apically, largely straight, apex narrowly rounded, surface imbricate, basal half with shallow, coarse punctures.

Clypeus with small punctures more widely spaced than in male, separated by less than a puncture width, punctures variable in size placed apically, but rather uniform medially, integument between smooth. Mesoscutum with well-defined punctures separated by less than a puncture width anteriorly and laterally, slightly more widely spaced on disc and posteriorly, there separated by 0.5–1.5 times a puncture width; mesoscutellum with punctures separated by less than a puncture width, less frequently separated by as much as a puncture width, axilla with punctures separated by less than a puncture width.

Integument and pubescence as in male except dark brown on pygidial plate; second through fifth metasomal terga with more or less transverse to rounded lateral patches (Figs 2, 4).

**Figures 7–11.**
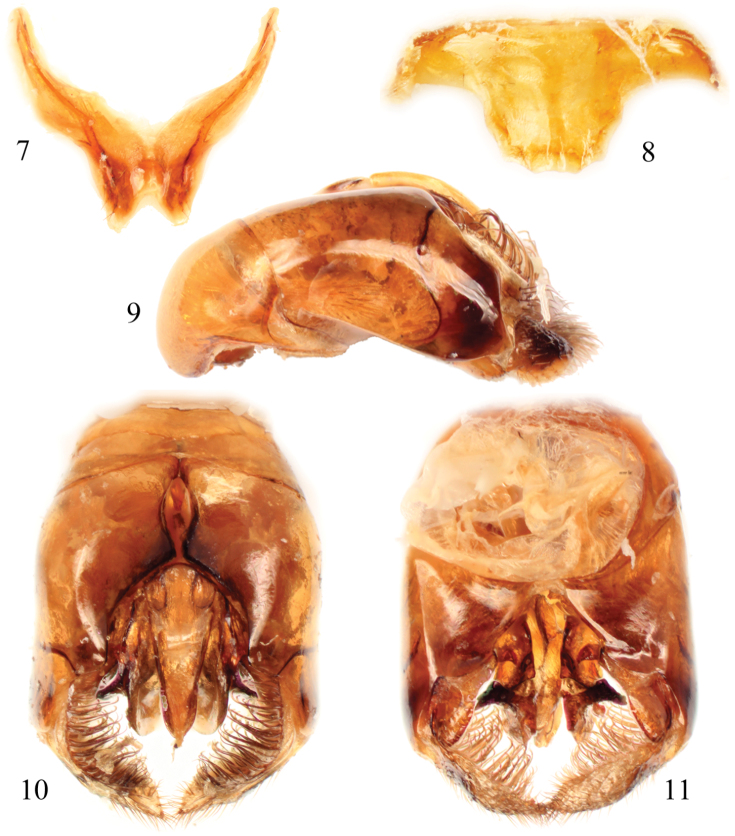
Male terminalia of *Thyreus denolii* sp. n. **7** Seventh metasomal sternum **8** Eighth sternum **9** Genital capsule, lateral aspect **10** Genital capsule, dorsal view **11** Genital capsule, ventral view.

##### Etymology.

The specific epithet is a patronym honoring António de Noli (ca. 1415–d. ?), a Genoese navigator who, exiled from his homeland and working on behalf of Portugal, discovered the Cape Verde Islands around 1456.

##### Comments.

The five females collected from Santiago are not designated as part of the type series. While they most closely agree with this species the shape of the pygidial plate is slightly different and this series could possibly represent a separate species. However, given that this is the only fixed difference we can find at this time and that we lack males from Santiago, we have tentatively assigned these individuals to *Thyreus denolii* pending the discovery of additional material. Analogously, a single female from S. Nicolau that is clearly of *Thyreus denolii* is also not designated as part of the type series. This might be a mislabeled specimen, a normal part of *Thyreus denolii*’s distribution, or even a different species. This particular female specimen of *Thyreus denolii* is unique in its L-shaped white patch on the second tergum. More material and collecting are needed so as to permit a more accurate characterization of the distribution and possible variation within the species.

#### Thyreus batelkai sp. n.

urn:lsid:zoobank.org:act:97D31AFF-AFE9-4234-8B7D-785D2E7B8020

http://species-id.net/wiki/Thyreus_batelkai

**Figures 12–13.**
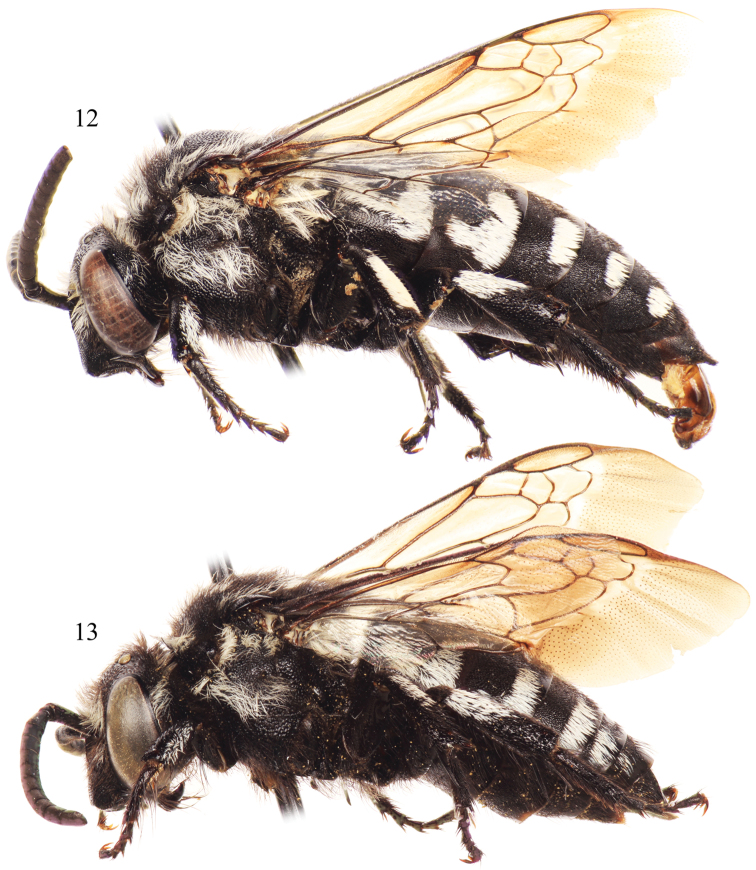
Lateral habitus of *Thyreus batelkai* sp. n. from Santo Antão. **12** Male **13** Female.

Figs 12 [Fig F7] –20

##### Holotype.

♂, Cape Verde Isl., Santo Antao, 1382 m, Espongeiro, 13.x.2009 [13 October 2009], 17°06'17"N, 25°05'21"W, J. Straka & J. Batelka lgt. (SEMC).

##### Paratypes.

**Santo Antão:** 1♀, 21.11.1980 [21 Noveber 1980], SANTO ANTAO, 400–1000 supra Porto Novo, Islas do Cabo Verde – 1980, H. Hölzel, W. Lobin, P. Ohm [collectors] (FISC); 1♀, Cabo Verde, Santo Antão, 5.01.99 [5 January 1999], Ribeira Grande, Orgãos, 50–200 m, leg. Aistleitner (EAFC); 1♀, Cabo Verde 00/10, Ilha d. S. Antão, Ribeira Grande, 2.12.2000 [2 December 2000], leg. Aistleitner (EAFC); 2♀♀, Cabo Verde 00/21, Ilha d, S. Antão, Cruzinha da Garça, 50 m, 7.12.2000 [7 December 2000], leg. Aistleitner (EAFC); 5♀♀, Cabo Verde 00/23, Ilha de S. Antão, Cruzinha da Garça, 10–50 m, 9.12.2000 [9 December 2000], leg. Aistleitner (EAFC); 1♂, 2♀♀, Cabo Verde 00/24, Ilha de S. Antão centr., Lagoa–E, 1150–1300 m, 10.12.2000 [10 December 2000], leg. Aistleitner (EAFC); 2♀♀, 1♂, Cape Verde Isl., Santo Antao, 1382 m, Espongeiro, 13.X.2009 [13 October 2009], 17°06'17"N, 25°05'21"W, J. Straka & J. Batelka lgt. (JSPC); 2♀♀, 1♂, same data as previous except 12.–13.X.2009 [12–13 October 2009], 17°06'N, 25°05'W (JSPC, SEMC); 8♀♀, 4♂♂, Cape Verde Isl., Santo Antao – Selada de Alto Mira – Cirio, 4.–5.XI.2011 [4–5 November 2011], 17°04'N, 25°13'W, J. Straka & J. Batelka lgt. (JSPC, FISC); 2♀, 1♂, Cape Verde Isl., Santo Antao – Curral das Vacas – Bordeira de Norte, 6.XI.2011 [6 November 2011], 17°02'N, 25°14'W, J. Batelka & J. Straka lgt. (JSPC); 1♂, Cape Verde Isl., Santo Antao – black dunes 3 km W of Porto Novo, 7.XI.2011 [7 November 2011], 17°0'47"N, 25°06'30.84"W, J. Straka & J. Batelka lgt. (JSPC); 1♀, Santo Antao, Selada de Alto Mira – Cirio, 4–5.xi.2011 [4–5 November 2011], 17°04'N, 25°13'W, J. Straka & J. Batelka lgt. (SEMC); **São Vicente:** 6♂♂, 10♀♀, Cabo Verde 00/25, Ilha de S. Vicente, Calhau – Baia d. Gatas, -5 m, 13.12.2000 [13 December 2000], leg. Aistleitner (EAFC).

##### Diagnosis

**Figures 14–15. F7:**
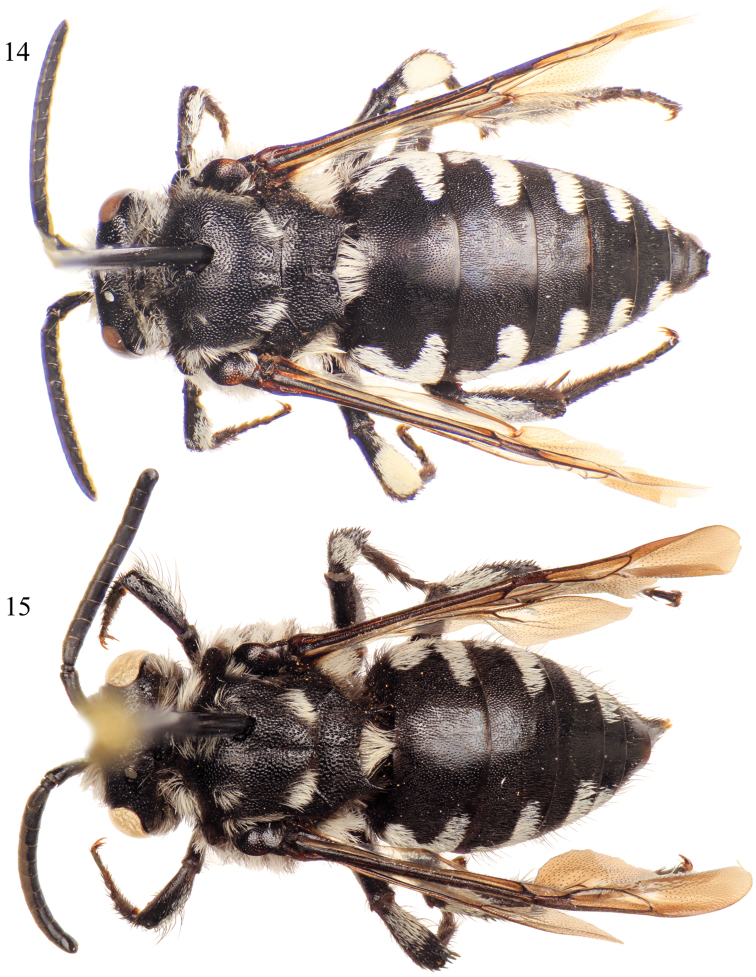
Dorsal habitus of *Thyreus batelkai* sp. n. from Santo Antão. **14** Male **15** Female.

**.** *Thyreus batelkai* can be distinguished in the male by the combination of the lateral white patches of the sixth metasomal tergum greatly reduced, especially in comparison to the patches on the fourth and fifth terga, and the second tergum with the lateral white patch L-shaped (as on the first tergum) or at least with a smaller anterior patch (Fig. 14); while the female can be recognized by the mesoscutum with *plsa* (anterior posterolateral mesoscutal) present anterior to and along the border with the tegula, extending posteriorly and meeting, or less frequently almost meeting, *pls* (posterolateral mesoscutal); the second metasomal tergum with the lateral white patch L-shaped (as on the first tergum) or at least with a smaller anterior patch (Figs 12, 14); and the apical depression of the fifth tergum bare medially, rarely with a few punctures with short setae. In both sexes the mesoscutum has coarse punctures throughout; the mesoscutellum is coarsely punctate, with punctures dense and nearly contiguous; the apicolateral corners of the mesoscutellum are prominently and sharply pointed, for-ming an angle of less than 40° (Figs 14, 15); and all of the white, anterior mesoscutal patches are well developed.

##### Description.

**Figures 16–20.**
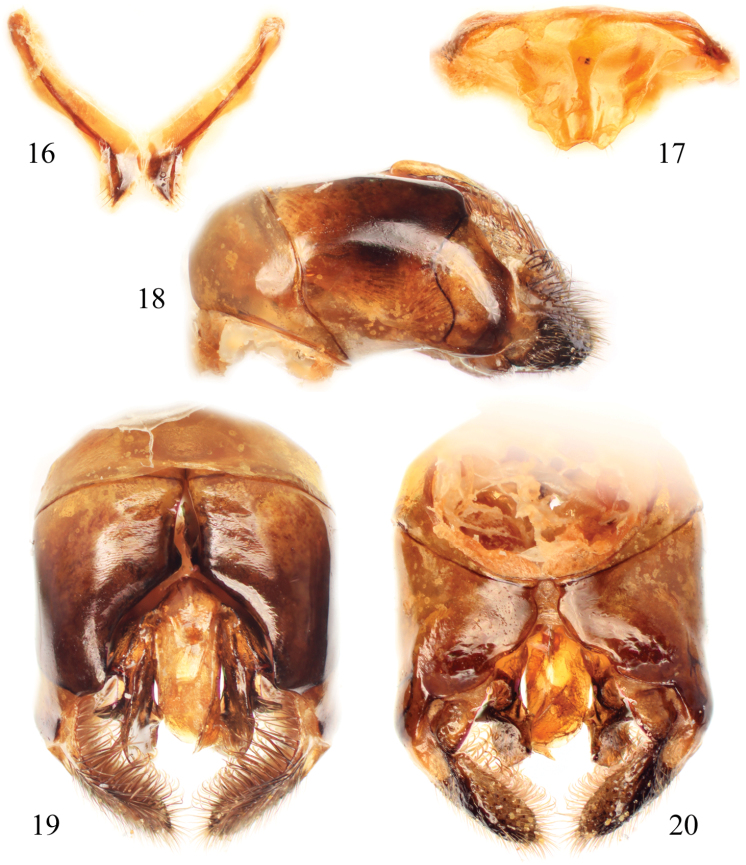
Male terminalia of *Thyreus batelkai* sp. n. **16** Seventh metasomal sternum **17** Eighth sternum **18** Genital capsule, lateral aspect **19** Genital capsule, dorsal view **20** Genital capsule, ventral view.

As described for *Thyreus denolii* (*vide supra*) except as follows: ♂: Total body length 13.7 mm (7.9–13.7 mm); forewing length 9.6 mm (6.5–10.6 mm). Head wider than long (length 2.7 mm, width 3.5 mm); upper interorbital distance 2.2 mm; lower interorbital distance 1.6 mm. Intertegular distance 3.1 mm (2.3–3.4 mm); mesoscutellar posterior margin weakly sinuate, with apicolateral angle projecting as a prominent spine, with a defined median emargination (Fig. 15). Apex of seventh metasomal tergum with apicolateral prominences distinct, margin between frequently broadly U-shaped, weakly concave, without medial emargination or swelling; male terminalia as in Figures 16–20.

Mesoscutum with well-defined, coarse, contiguous to nearly contiguous punctures; mesoscutellum, including axilla, as on mesoscutum. Pleura with coarse, contiguous punctures; hypoepimeral area as on remainder of pleura, without impunctate area bordering scrobe.

White patches on mesosoma as follows: *deps* (dorsal mesepisternal), *lpn* (lateral pronotal), and *als* (anterolateral mesoscutal) present; *ms* (median mesoscutal) present, often extending to posterior tangent of *mls* (mediolateral mesoscutal); *mls* (mediolateral mesoscutal) present; *plsa* (anterior posterolateral mesoscutal) present anterior to and along border with tegula, extending posteriorly and meeting, or less frequently almost meeting, *pls* (posterolateral mesoscutal); *t* (tegular) present posteriorly on tegula; *pls* (posterolateral mesoscutal) present; *ps* (parascutellar) and *s* (mesoscutellar) absent; *deps* (dorsal mesepisternal), *hypm* (hypoepimeral area), and *lp* (lateral propodeal) present, *veps* (ventral mesepisternal) absent (Figs 12, 14). Metasomal terga with prominent patches of appressed, plumose white setae as follows: first metasomal tergum with large, L-shaped patches laterally; second metasomal tergum with large, L-shaped patches laterally as on first tergum, infrequently L-shape is incomplete with secondary rounded secondary anterior patch almost connecting posterior transverse patch; third through fifth metasomal terga with more or less transverse to rounded lateral patches (Figs 12, 14).

♀: As described for the male except in usual gender differences and as follows: Total body length 7.4–13.6 mm; forewing length 5.7–10.4 mm. Head wider than long (length 3.2 mm, width 3.9 mm); upper interorbital distance 2.3 mm; lower interorbital distance 1.8 mm. Intertegular distance 1.7–3.4 mm; mesoscutellar posterior margin forming a more gently, broad curve that is more straight medially between apicolateral angles, apicolateral angles forming a prominent spine, medially not to faintly emarginate. Apical depression of fifth tergum impunctate medially. Pygidial plate relatively narrow, margins converging apically, largely straight, apex narrowly rounded, surface imbricate except apicolaterally with smooth areas, basal half with shallow, coarse punctures.

Clypeus with small punctures separated by less than a puncture width, but with variable punctures in size apically and more sparsely punctate than in middle, some interspaces among punctures about as large as a puncture width, integument between smooth.

Integument and pubescence as in male except dark brown on pygidial plate; third through fifth metasomal terga with more or less transverse to rounded lateral patches (Figs 13, 15).

##### Etymology.

The specific epithet is a patronym honoring Jan Batelka, a prominent collector of the new species, authority on the systematics of beetles, and close friend.

#### Thyreus schwarzi sp. n.

urn:lsid:zoobank.org:act:92D8F800-D36A-4B7E-AADE-BA3F770B96DD

http://species-id.net/wiki/Thyreus_schwarzi

**Figures 21–22.**
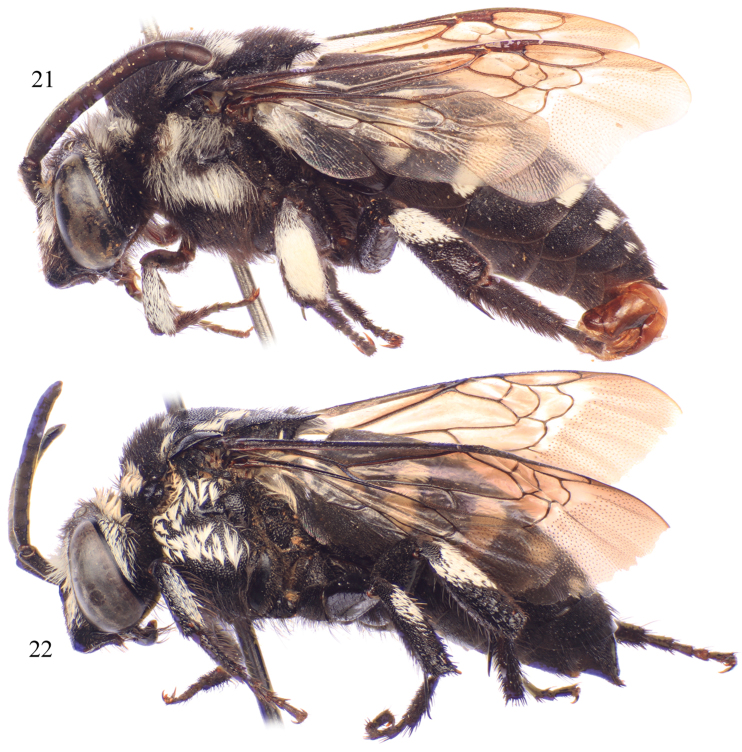
Lateral habitus of *Thyreus schwarzi* sp. n. from São Nicolau. **21** Male **22** Female.

Figs 21 [Fig F10] –29

##### Holotype.

♂, Isl., Cabo Verde, S. Nicolau, 1.XII.80 [1 December 1980], Ribeiro Brava, Islas do Cabo Verde - 1980, H. Hölzel, W. Lobin, P. Ohm [collectors] (FISC).

##### Paratypes.

**São Nicolau:** 1♂, same data as holotype (FISC); 1♀, Ilhas do Cabo Verde, S. Nicolau, Lobin leg. (FISC); 1♀, Cabo Verde 00/31, Ilha de S. Nicolau, Ribeira Brava–W, 100–150 m, 20.12.2000 [20 December 2000], leg. Aistleitner (EAFC); 3♂♂, Cabo Verde 00/34, Ilha de S. Nicolau, Preguiça–N, 70–100 m, 21.12.2000 [21 December 2000], leg. Aistleitner (EAFC); 4♂♂, Cabo Verde 00/36, Ilha de S. Nicolau, Monte Gordo, 12–1300 m, 22.12.2000 [22 December 2000], leg. Aistleitner (EAFC); 1♂, Cape Verde Isl., Sao Nicolau W, Barril, 11.XI.2011 [11 November 2011], 16°35'24"N, 24°23'84"W, J. Straka & J. Batelka lgt. (JSPC); 1♀, Sao Nicolau, W, south of Cachao, Cha de Caldeira, 12.xi.2011, 16°36'39"N, 24°19'58"W, J. Batelka & J. Straka lgt. (SEMC).

##### Additional material.

**Santo Antão:**1♀, Cabo Verde 00/21, Ilha d, S. Antão, Cruzinha da Garça, 50 m, 7.12.2000 [7 December 2000], leg. Aistleitner (EAFC).

##### Diagnosis.

**Figures 23–24. F10:**
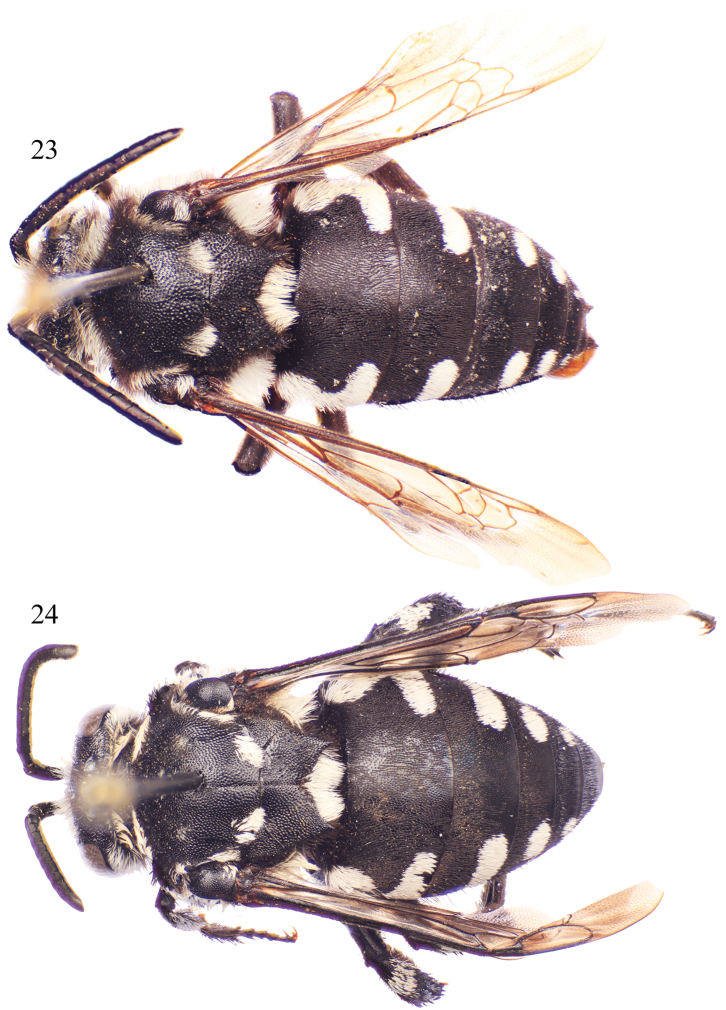
Dorsal habitus of *Thyreus schwarzi* sp. n. from São Nicolau. **23** Male **24** Female.

Males of *Thyreus schwarzi* can be recognized by the following combination of characters: white anterior mesoscutal patches, especially *ms* (median mesoscutal) and *mls* (mediolateral mesoscutal), strongly reduced to missing (Fig. 23); second metasomal tergum with lateral patch wider than long, without anterior secondary patch and never L-shaped (Fig. 23), but rarely with a few white setae in this area; and sixth tergum with lateral white patches greatly reduced, especially in comparison to patches on the fourth and fifth terga. Females can be characterized by the following combination of traits: white anterior mesoscutal patches, especially paired *mls* (mediolateral mesoscutal), greatly reduced to a few short setae (Fig. 24); mesoscutum with *plsa* (anterior posterolateral mesoscutal) present anterior to and along border with tegula, extending posteriorly and meeting, or less frequently almost meeting, *pls* (posterolateral mesoscutal) (Fig. 24); second metasomal tergum with lateral patch wider than long, without anterior secondary patch and never L-shaped (Fig. 24), but rarely with a few white setae in this area; and apical depression of fifth tergum with several distinct seta-bearing punctures. Both males and females have the apicolateral corners of the mesoscutellum prominently and sharply pointed, forming an angle of less than 40° (Figs 23, 24)

##### Description.

**Figures 25–29.**
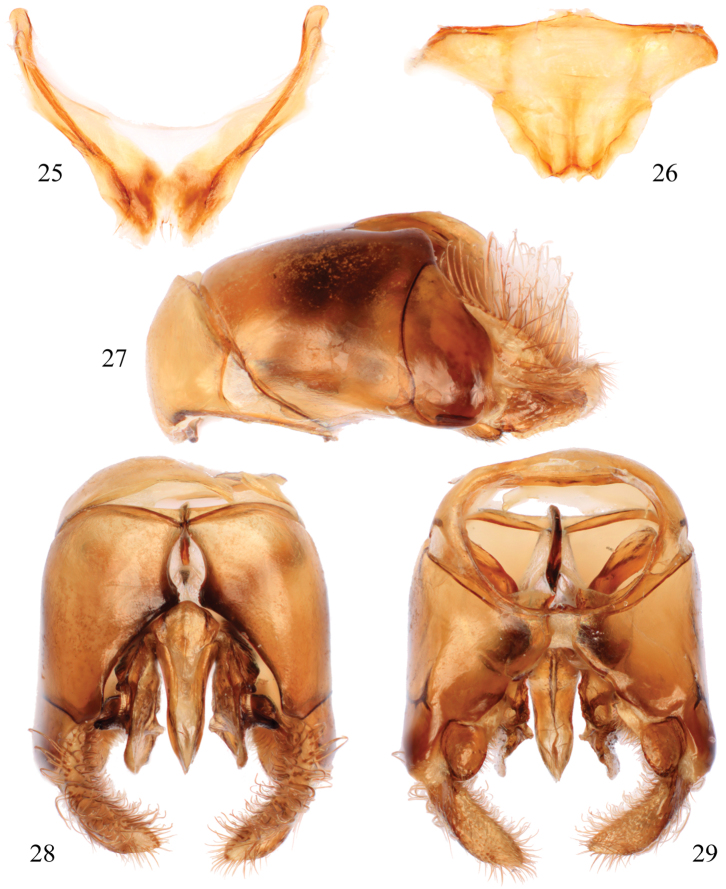
Male terminalia of *Thyreus schwarzi* sp. n. **25** Seventh metasomal sternum **26** Eighth sternum **27** Genital capsule, lateral aspect **28** Genital capsule, dorsal view **29** Genital capsule, ventral view.

As described for *Thyreus denolii* (*vide supra*) except as follows: ♂: Total body length 12.8 mm (8.5–14.0 mm); forewing length 10.4 mm (6.7–10.5 mm). Head wider than long (length 3.1 mm, width 3.7 mm); upper interorbital distance 2.3 mm; lower interorbital distance 1.7 mm. Intertegular distance 3.4 mm (2.1–3.5 mm); mesoscutellar posterior margin faintly sinuate, with apicolateral angle projecting as slightly prominent spine, with median emargination. Apex of seventh metasomal tergum with apicolateral prominences distinct, truncate margin between straight, without medial emargination or swelling; male terminalia as in figures 25–29.

Mesoscutum with well-defined punctures separated by much less than a puncture width, punctures mediopically more spaced, separated by about 0.5–1 times a puncture width but more often less than a puncture width, integument between punctures smooth and shining; mesoscutellum, including axilla, with punctures separated by less than a puncture width, those laterally nearly contiguous to contiguous.

White patches on mesosoma as follows: *deps* (dorsal mesepisternal) and *lpn* (lateral pronotal) present; *als* (anterolateral mesoscutal) present but reduced, often faint; *ms* (median mesoscutal) faint to absent; *mls* (mediolateral mesoscutal) present to faint; *plsa* (anterior posterolateral mesoscutal) present anterior to and along border with tegula, extending posteriorly and meeting, or less frequently almost meeting, *pls* (posterolateral mesoscutal); *t* (tegular) present and prominent posteriorly on tegula; *pls* (posterolateral mesoscutal) present; *ps* (parascutellar) and *s* (mesoscutellar) absent; *deps* (dorsal mesepisternal), *hypm* (hypoepimeral area), and *lp* (lateral propodeal) present, *veps* (ventral mesepisternal) absent (Figs 21, 23). Metasomal terga with prominent patches of appressed, plumose white setae as follows: first metasomal tergum with large, L-shaped patches laterally; second metasomal tergum with lateral patch transverse, slightly wider than twice as long, never L-shaped and without rounded secondary anterior patch; third through fifth metasomal terga with more or less transverse to rounded lateral patches (Figs 21, 23).

♀: As described for the male except in usual gender differences and as follows: Total body length 11.8–13.1 mm; forewing length 9.4–10.2 mm. Head wider than long (length 3.0 mm, width 3.8 mm); upper interorbital distance 2.3 mm; lower interorbital distance 1.7 mm. Intertegular distance 2.8–3.3 mm; mesoscutellar posterior margin as in male but sinuate margin stronger. Apical depression of fifth tergum sparsely but distinctly punctate and setose. Pygidial plate relatively broad, margins converging apically, largely straight, apex narrowly rounded, surface imbricate, basal half with shallow, coarse punctures.

Mesoscutum with well-defined punctures separated by much less than a puncture width, punctures mediopically slightly more spaced but still separated by less than a puncture width; mesoscutellum, including axilla, with punctures separated by less than a puncture width.

Integument and pubescence as in male except dark brown on pygidial plate; second through fourth metasomal terga with more or less transverse to rounded lateral patches (Figs 22, 24).

##### Etymology.

The specific epithet is a patronym honoring Dr. Maximilian Schwarz, a leading authority on the systematics of cuckoo bees and a dear colleague.

##### Comments.

A single female collected from Santo Antão is not designated as part of the type series since the specimen may be mislabeled or a rare case of introduction to a different island.

#### Thyreus aistleitneri sp. n.

urn:lsid:zoobank.org:act:B992FB5F-C606-4CA7-BE75-5021D4AE36F7

http://species-id.net/wiki/Thyreus_aistleitneri

Figs 30 –34

##### Holotype.

♀, Cabo Verde, Brava, Nova Sintra, Mte. Nha Preta, 700–880 m, 25.01.01 [25 January 2001], leg. Aistleitner (SEMC).

##### Diagnosis.

**Figures 30–31.**
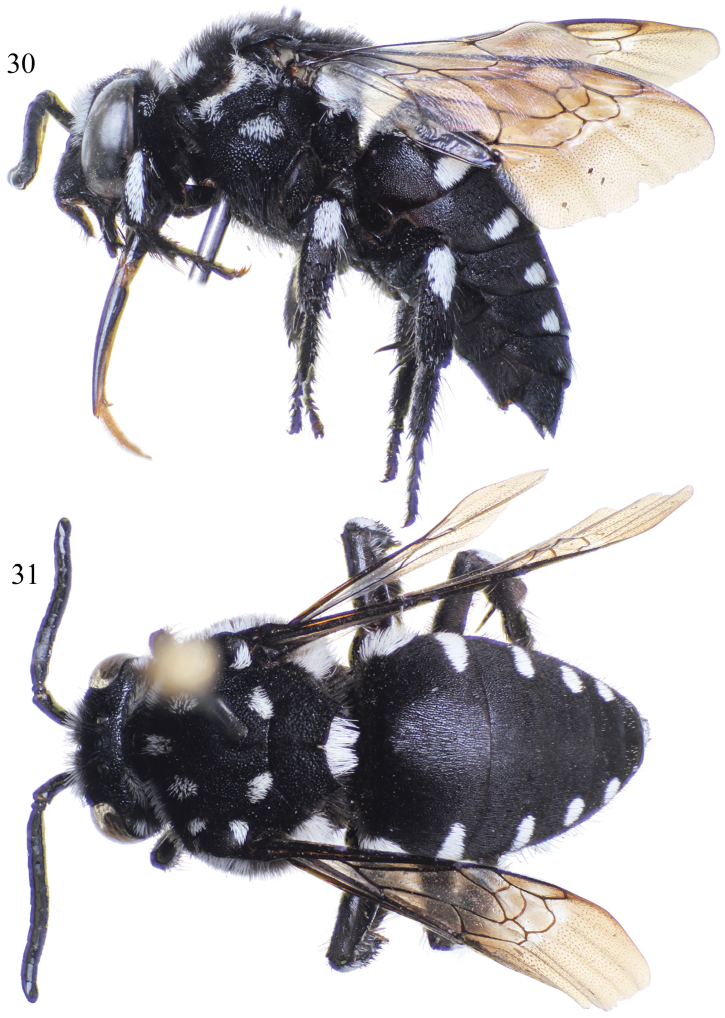
Female of *Thyreus aistleitneri* sp. n. from Brava. **30** Lateral habitus **31** Dorsal habitus.

The new species can be recognized by the following combination of features: mesoscutum with *plsa* (anterior posterolateral mesoscutal) present and bordering anterior portion of tegula, not meeting *pls* (posterolateral mesoscutal) posteriorly (Fig. 31); mesoscutellum coarsely punctate, with punctures dense, separated, at least on disc, by less than 0.5 times a puncture width; apicolateral corners of mesoscutellum weakly pointed, forming angle of more than 40° (Fig. 33); ventral and ventrolateral pleura with distinct shiny interspaces among punctures; apical depression of fifth metasomal tergum with a few isolated punctures medially; fifth tergum with lateral white patches reduced, especially in comparison to patches on fourth and third tergum (Figs 30, 31, 34).

##### Description.

**Figures 32–34.**
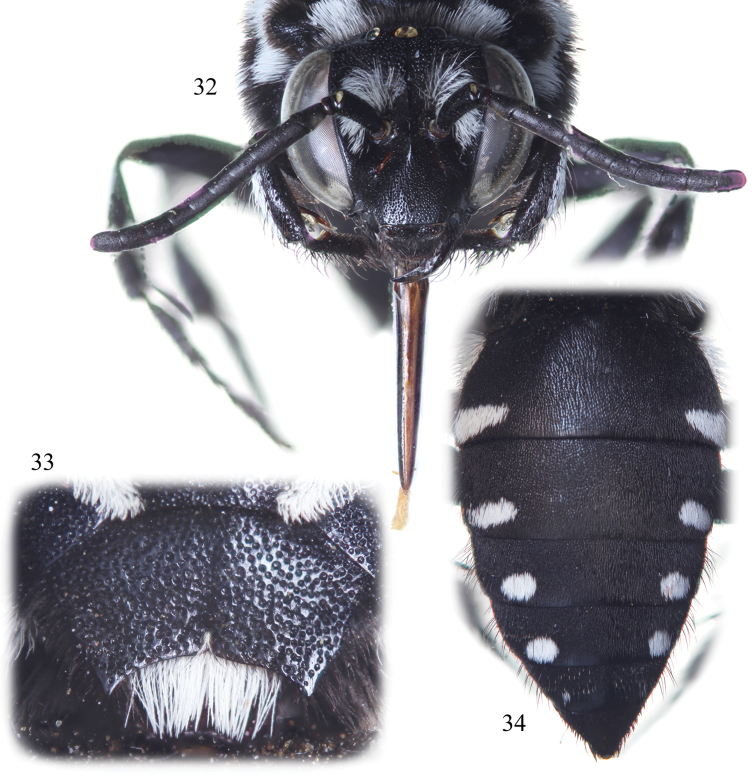
Female of *Thyreus aistleitneri* sp. n. from Brava. **32** Facial aspect **33** Detail of mesoscutellum **34** Detail of metasoma in dorsal view.

As described for *Thyreus denolii* (*vide supra*) except as follows: ♀: Total body length 11.3 mm; forewing length 8.3 mm. Head wider than long (length 2.9 mm, width 3.5 mm) (Fig. 32); upper interorbital distance 2.1 mm; lower interorbital distance 1.55 mm. Intertegular distance 2.7 mm; mesoscutellar posterior margin faintly sinuate, apicolateral angle projects as a prominent, broad spine and with a defined median emargination (Figs 31, 33). Apical depression of fifth tergum sparsely, but distinctly, punctate and setose. Pygidial plate relatively narrow, margins converging apically, slightly sinuate, apex narrowly rounded, surface imbricate, basal half with shallow, coarse punctures.

Mesoscutum with well-defined punctures separated by much less than a puncture width, punctures mediopically more spaced, separated by about 0.5–1 times a puncture width but more often less than a puncture width, integument between punctures smooth and shining; mesoscutellum, including axilla, with punctures separated by less than a puncture width, those laterally nearly contiguous (Fig. 33).

White patches on mesosoma as follows: *deps* (dorsal mesepisternal) and *lpn* (lateral pronotal) present; *als* (anterolateral mesoscutal) present but reduced; *ms* (median mesoscutal) faint; *mls* (mediolateral mesoscutal) present; *plsa* (anterior posterolateral mesoscutal) present along anterior half of border with tegula, not meeting *pls* (posterolateral mesoscutal) posteriorly; *t* (tegular) present and prominent posteriorly on tegula; *pls* (posterolateral mesoscutal) present, not extending laterally to meet *plsa* (anterior posterolateral mesoscutal); *ps* (parascutellar) and *s* (mesoscutellar) absent; *deps* (dorsal mesepisternal), *hypm* (hypoepimeral area), and *lp* (lateral propodeal) present, *veps* (ventral mesepisternal) absent (Fig. 31). Pleural white spot reduced to two separate spots (Fig. 30). Metasomal terga with prominent patches of appressed, plumose white setae as follows: first metasomal tergum with L-shaped patch laterally interrupted in middle and thus divided into two spots, one transverse at apicolateral margin and one triangular on tergal side; second metasomal tergum with reduced lateral patch transverse, slightly wider than twice as long, without rounded secondary anterior patch; third and fourth metasomal terga with rounded lateral patches; fifth metasomal tergum with lateral spot reduced to a few setae (Figs 30, 31, 34); pygidial plate with dark brown setae.

♂: Unknown.

##### Etymology.

The specific epithet is a patronym honoring Eyjolf Aistleitner, collector of the new species among many fine insects, and authority on the systematics of Lepidoptera.

### Key to the Species of *Thyreus* of the Cape Verde Islands

**Table:** 

1	Males	2
–	Females	4
2	Sixth metasomal tergum with lateral white patches of the same size as on fourth and fifth terga; mesoscutellum finely punctate, with punctures separated, at least on disc, by 0.5–1 times a puncture width; apicolateral corners of mesoscutellum weakly pointed (angle more than 40°); metasoma densely punctate, punctures reach almost the end of metasomal terga (Boavista, Sal, Santiago*, S. Nicolau**)	*Thyreus denolii* sp. n.
–	Sixth metasomal tergum with lateral white patches greatly reduced, especially in comparison to patches on fourth and fifth tergum; mesoscutellum more coarsely punctate, with punctures dense, nearly contiguous; apicolateral corners of mesoscutellum more prominently and sharply pointed (angle less than 40°); metasoma more sparsely punctate, punctures separated from margins	3
3	Second metasomal tergum with lateral white patch L-shaped (as on first tergum) or at least with smaller anterior patch (Fig. 14); mesoscutum with coarse punctures throughout; all white anterior mesoscutal patches well developed (S. Antão)	*Thyreus batelkai* sp. n.
–	Second metasomal tergum with lateral patch wider than long, without anterior secondary patch and never L-shaped (Fig. 23), but rarely with few white setae in this area; mesoscutellum with finer punctures at least basomedially; white anterior mesoscutal patches, especially *ms* (median mesoscutal) and *mls* (mediolateral mesoscutal), strongly reduced to missing (Fig. 23) (S. Nicolau)	*Thyreus schwarzi* sp. n.
4	Apicolateral corners of mesoscutellum weakly pointed (angle more than 40°) (e.g., Fig. 33); mesoscutellum finely punctate, with punctures separated, at least on disc, by 0.5–1 times a puncture width (except *Thyreus aistleitneri*); ventral and ventrolateral portions of pleura with distinct shiny interspaces among punctures; mesoscutum with *plsa* (anterior posterolateral mesoscutal) present and bordering anterior portion of tegula, not meeting *pls* (posterolateral mesoscutal) posteriorly (e.g., Figs 4, 31)	5
–	Apicolateral corners of mesoscutellum sharply pointed (angle less than 40°) (Figs 15, 24); mesoscutellum more coarsely punctate, with punctures dense, nearly contiguous; pleura ventrally and ventrolaterally coarsely punctate, interspaces among most punctures indistinct (smallest specimens sometimes with sparsely punctate pleura); mesoscutum with *plsa* (anterior posterolateral mesoscutal) present anterior to and along border with tegula, extending posteriorly and meeting, or less frequently almost meeting, *pls* (posterolateral mesoscutal) (e.g., Figs 15, 24)	6
5	Apical depression of fifth tergum densely punctate medially and densely setose; fifth metasomal tergum with lateral white patches of same size as on fourth and third terga (Fig. 4) (rarely white patches on fifth tergum reduced, but then reduced also on preceding terga); mesoscutellum finely punctate, with punctures separated, at least on disc, by 0.5–1 times a puncture width (Boavista, Sal, Santiago*, S. Nicolau**)	*Thyreus denolii* sp. n.
–	Apical depression of fifth tergum with a few isolated punctures medially; fifth metasomal tergum with lateral white patches reduced, especially in comparison to patches on fourth and third terga (Figs 30, 31, 34); mesoscutellum more coarsely punctate, with punctures dense, separated, at least on disc, by less than 0.5 times a puncture width (Fig. 33) (Brava)	*Thyreus aistleitneri* sp. n.
6	Apical depression of fifth tergum bare medially, rarely with few punctures with short seta; second metasomal tergum with lateral white patch L-shaped (as on first tergum) or at least with smaller anterior patch (Fig. 15); mesoscutum with coarse punctures throughout; all white anterior mesoscutal patches well developed (Fig. 15) (Santo Antão)	*Thyreus batelkai* sp. n.
–	Apical depression of fifth tergum with several distinct seta-bearing punctures; second metasomal tergum with lateral patch wider than long, without anterior secondary patch and never L-shaped (Fig. 24), but rarely with few white setae in this area; mesoscutellum with finer punctures at least basomedially; white anterior mesoscutal patches, especially paired *mls* (mediolateral mesoscutal), greatly reduced to a few short setae (São Nicolau)	*Thyreus schwarzi* sp. n.

* The Santiago females of *Thyreus denolii* are much larger, probably because of the larger host, and differ slightly in the shape of the pygidial plate. We presently interpret this as merely minor variations in the absence of more conclusive evidence suggesting a separate species status for the Santiago population. ** It seems unusual that there is a single female from S. Nicolau that is clearly of *Thyreus denolii*. Refer to the account for that species regarding this single individual which is perhaps mislabeled. However, this specimen of *Thyreus denolii* is unique for its L-shaped white patch on the second tergum. More material is needed in order to permit a more thorough understanding of their variability.

### Subfamily Nomadinae Latreille. Tribe Ammobatini Handlirsch. Genus *Chiasmognathus* Engel

The genus *Chiasmognathus* comprises a series of small ammobatine bee species which are exclusively cleptoparasitic in the nests of Nomioidini (Engel 2006, 2007, 2008a, 2008b, 2009, 2010). Although few species have been characterized, the genus is likely quite diverse perhaps with numerous undescribed species occurring wherever there are nesting aggregations of *Nomioides* Schenck or *Ceylalictus* Strand. The biology and immature stages of a single species have been studied in Pakistan (Rozen 2008). The discovery of the new species described here brings the described diversity of the genus up to 11 species, the new one being the most westerly of the known taxa, although new species are being discovered semi-regularly (Engel pers. obs.). *Chiasmognathus batelkai* appears to victimize nests of *Chiasmognathus capverdensis*. Vouchers of the host species can be found in the EAFC, SEMC, NMPC, and JSPC. Interestingly, even at 3.2–4.2 mm in length, *Chiasmognathus batelkai* represents a case of island ‘gigantism’ as this is the largest known species of the genus.

#### Chiasmognathus batelkai sp. n.

urn:lsid:zoobank.org:act:4ED85B90-4C8B-47B7-B96D-CF4A7061A7C0

http://species-id.net/wiki/Chiasmognathus_batelkai

**Figures 35–36. F14:**
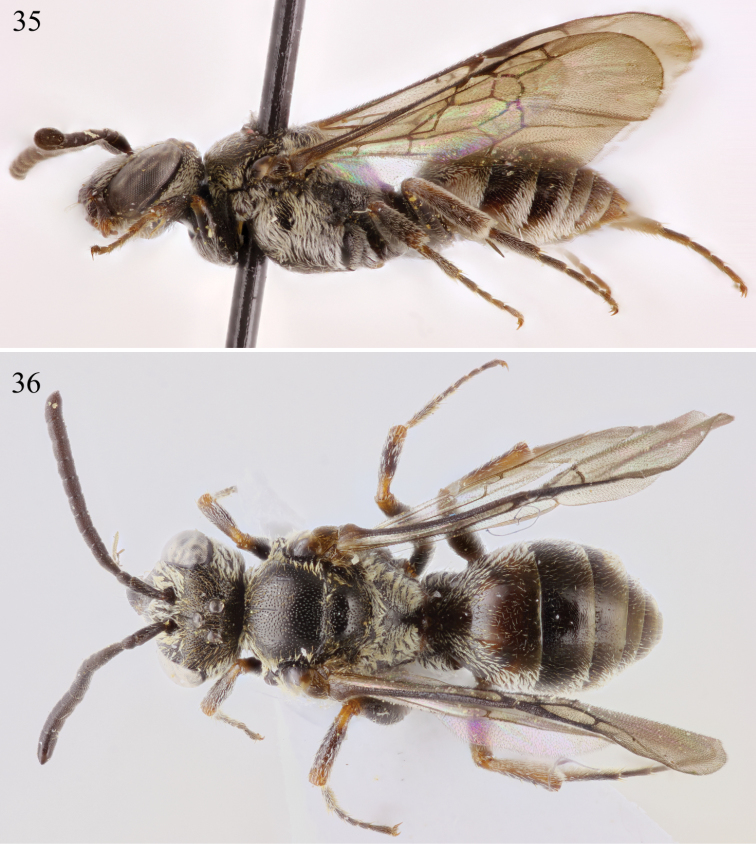
Male habitus of *Chiasmognathus batelkai* sp. n. **35** Lateral **36** Dorsal.

Figs 35 [Fig F14] [Fig F15] –43

##### Holotype.

♂, Cape Verde Isl., Santo Antao, 1382 m, Espongeiro, 13.X.2009 [13 October 2009], 17°06'17"N, 25°05'21"W, J. Straka & J. Batelka lgt. (SEMC).

##### Paratypes.

**São Vicente:** 1♂, Cabo Verde 00/25, Ilha de S. Vicente, Calhau – Baia d. Gatas, -5 m, 13.12.2000 [13 December 2000], leg. Aistleitner (EAFC); **Santo Antão:** 5♂♂, same data as holotype (FISC, JSPC, SEMC); 3♂♂, 4♀♀, same data as holotype except 12–13.X.2009 [12–13 October 2009], 17°06'N, 25°05'W (JSPC, SEMC).

##### Diagnosis.

The new species is the largest of the known *Chiasmognathus*, being 3.2–4.1 mm in total length in females and 3.5–4.2 mm in males. The ocellar elevation in males is distinctly more prominent than in any of the other species, this and the combination of the size, mesoscutal sculpturing, and coloration (Figs 35, 36, 42, 43) will serve to identify *Chiasmognathus batelkai* from the other species of the genus, particularly those occurring in Africa (Engel 2010, pers. obs.: Niger and undescribed species from Kenya).

##### Description.

**Figures 37–41. F15:**
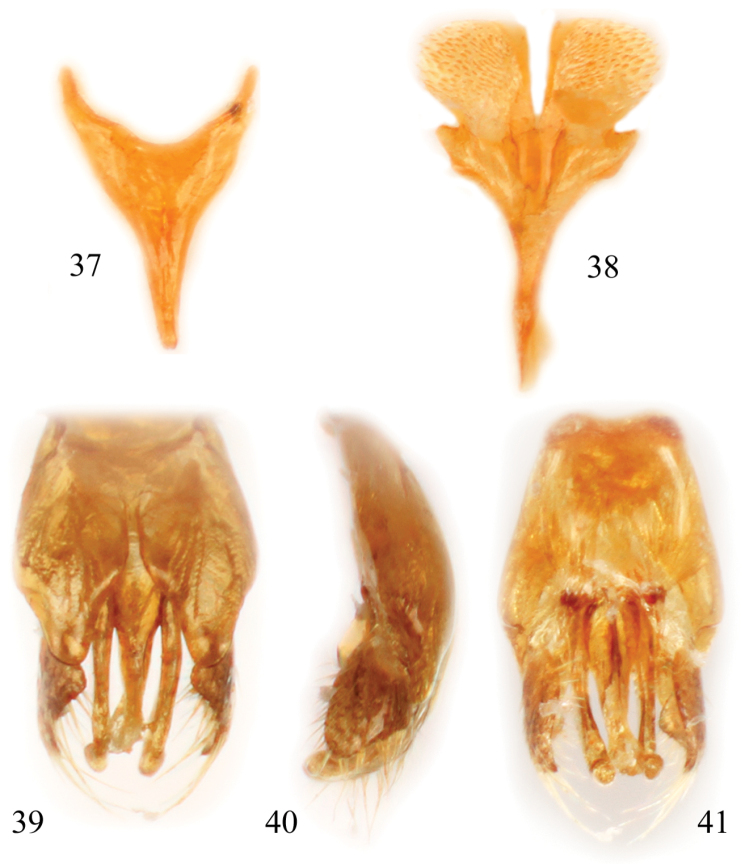
Male terminalia of *Chiasmognathus batelkai* sp. n. **37** Seventh metasomal sternum **38** Eighth sternum **39** Genital capsule, dorsal view **40** Genital capsule, lateral aspect **41** Genital capsule, ventral view.

♂: Total body length 4.0 mm (3.5–4.2 mm); forewing length 3.3 mm (3.1–3.7 mm). Head wider than long (width 1.3 mm, length 0.99 mm); inner margins of compound eyes straight, convergent below; apex of clypeus at lower tangent of compound eyes; ocelli above upper tangent of compound eyes, ocellar triangle particularly prominent, swollen above curvature of head (more so than in other species of the genus); clypeus weakly convex, nearly flat, apicolateral corners of clypeus with small patches of tightly packed, elongate, apically-sinuate setae; malar space vestigial; mandibles simple, crossing in repose but not covering labrum; frontal line carinate from just below lower tangent of antennal toruli to median ocellus. Mesoscutum with median line deeply impressed and wide, width about that of mesoscutal puncture diameter, extending to just before mesoscutal midlength. Intertegular distance (i.e., distance between inner margins of tegulae) 0.8 mm (0.7–0.9 mm). Forewing marginal cell broadly truncate; both m-cu crossveins entering second submarginal cell. Male terminalia as depicted in Figures 37–41.

Integument generally shining and smooth (Fig. 36). Labrum with punctures over entire surface, punctures nearly contiguous, integument between punctures (where evident) smooth; clypeus with shallow punctures separated by 0.5–1.5 times a puncture width centrally, punctures separated by less than a puncture width laterally; face and vertex with punctures nearly contiguous and more well defined than those centrally on clypeus, integument between (where evident) smooth, punctures on vertex posterior to ocelli somewhat weaker; punctures on gena as on sides of vertex with punctures gradually becoming more widely spaced ventrally; postgena with smaller and weaker punctures separated by 1–4 times a puncture width, integument otherwise smooth but duller than shining integument elsewhere on head. Mesoscutum punctate, anteriorly and around median line punctures separated by 0.5 times a puncture width, infrequently more widely spaced, otherwise punctures of mesoscutum separated by 1–2 times a puncture width, infrequently by less; mesoscutellum with punctures separated by 0.5 times a puncture width except in paramedial areas of disc distinctly more sparse, separated there by 1–5 times a puncture width; metanotum with punctures separated by less than a puncture width; preëpisternal area with punctures more coarse than those of meoscutum, nearly contiguous; hypoepimeral area with small punctures separated by 0.5–3 times a puncture width, ventrally with largely impunctate area bordering scrobe; mesepisternum with punctures separated by 0.5–2 times a puncture width anteriorly, posteriorly punctures more coarse and closer, often nearly contiguous, becoming more widely spaced ventrally; metepisternum with punctures separated by less than a puncture width above, punctures becoming more widely spaced ventrally; propodeum with short and narrow basal area coarsely imbricate and impunctate, otherwise integument with punctures separated by less than a puncture width. Metasomal terga and sterna finely imbricate, with scattered weak, small punctures, apical margins impunctate.

Integument of head and mesosoma black and shining (Figs 35, 36) except reddish brown on mandibular apex, brown on middle third of mandible, light brown on palpi and glossa, dark brown to black on labrum (some males with reddish brown laterally on labrum), dark brown to black on antennae, dark brown on tegula, and dark brown on legs except lighter on tarsi and at femorotibial and tibiobasitarsal joints. Wing veins brown except C and Sc+R dark brown; membranes hyaline, forewings faintly infumate. Metasoma dark brown except first tergum dark reddish brown in apical two-thirds to one-half; apical margins of terga narrowly brown to light brown.

Pubescence silvery white. Head with numerous, fine, appressed to subappressed plumose setae, such setae nearly obscuring integument of face around and below level of antennal toruli, and intermingled with a few suberect to erect finer, simple setae; such appressed plumose setae present on gena. Setae of mesosoma like those of head although more sparse centrally on mesoscutum and mesoscutellum; setae similar to those of gena on pleura (although longer and more diffuse to sparse centrally on mesepisternum), metanotum, and dorsolateral portions of propodeum. Metasoma with sparse, erect to suberect, short simple setae, without prominent apical fasciae composed of appressed, plumose, white pubescence; first metasomal tergum with small, weak apicolateral patches of appressed to subappressed plumose setae; succeeding terga with similar patches although often more diffuse or narrower than those of first tergum.

♀: As described for the male except in usual gender differences (Engel 2006, 2009) and as follows: Total body length 3.2–4.1 mm; forewing length 2.9–3.9 mm. Ocellar triangle not as prominent as in male. Intertegular distance 0.7–1.0 mm.

Metasomal terga and sterna finely imbricate, with sparse weak, minute punctures, apical margins impunctate, often broadly so.

**Figures 42–43.**
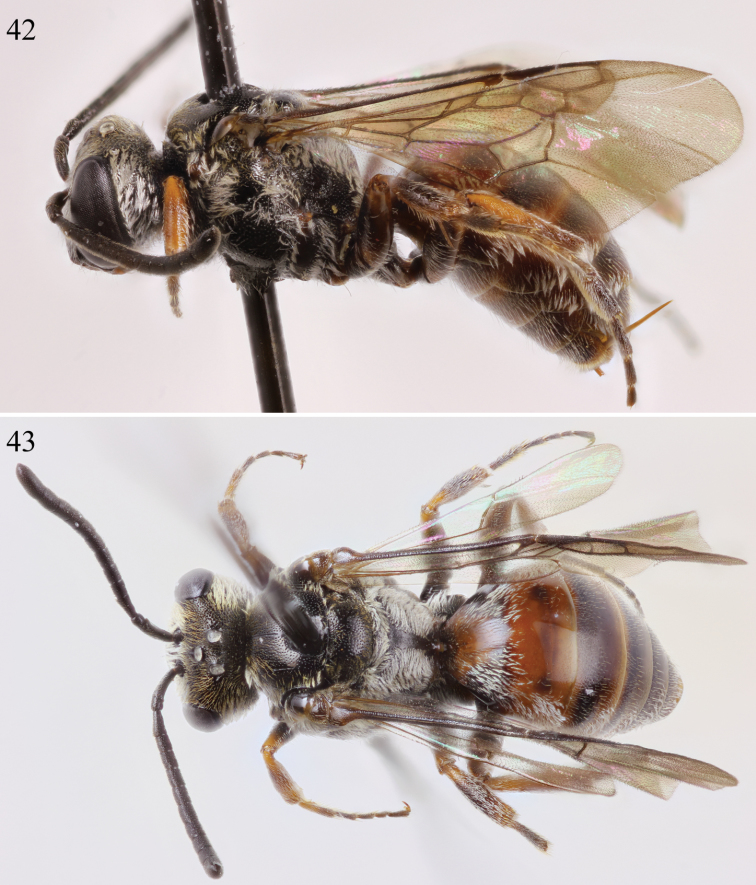
Female habitus of *Chiasmognathus batelkai* sp. n. **42** Lateral **43** Dorsal.

Coloration as in male except often protibia brown as on tarsi rather than dark brown as on other more basal podites; metasoma dark brown except first tergum largely reddish brown in apical two-thirds to one-half (Figs 42, 43); apical margins of terga reddish brown to brown, most prominently so on second tergum, less so on more apical terga and sterna.

##### Etymology.

The specific epithet is a patronym honoring Jan Batelka, a prominent collector of the new species, authority on the systematics of beetles, and good friend.

## Discussion

It appears as though the Cape Verde Islands fauna of *Thyreus* represents a single invasion of the archipelago with subsequent speciation across the islands. All these species resemble *Thyreus hohmanni* Schwarz endemic to the Canary Islands (another Macaronesian archipelago) and the more widespread *Thyreus ramosus*. It is possible that the *Thyreus* of Cape Verde came from the Canary Islands, and follow a similar pattern of relationship and introduction as is described for some plant species (Kim et al. 2008), and it is similarly probable that all endemic macaronesian *Thyreus* share a nearest common ancestor within populations of *Thyreus ramosus* s.l. and to the exclusion of other congenerics. It certainly appears as though the bees have moved among the islands, perhaps during sea level regressions when distances would have reduced, speciated and then again dispersed, with cycles of this giving the present distributions of the species (Map 1). It is interesting to note that although the populations of *Thyreus denolii* across Sal, Boavista, and Santiago are relatively isolated, they do not differ significantly in any observed morphological features. If all of these island populations do belong to the same species as we have treated them herein, then *Thyreus denolii* is parasitic on three different host species of *Amegilla* (Engel and Straka in prep.). Conversely, two different cuckoo bee species, *Thyreus batelkai* and *Thyreus schwarzi*, share identical host species. This suggests that the pattern of speciation in the Cape Verde *Amegilla*-*Thyreus* host-parasite complex is not a simple co-evolutionary scenario but instead reflects a complicated history intermingling factors such as probably different times of introduction of host (earlier) and parasite (later) populations, differential spread across the islands, and changing sea levels. The sea level between some islands is shallow (particularly the waters between Sal and Boavista, between the Barlavento islands, and among the Sotavento islands), and Sal and Boavista might have been connected in the geological past (Ramalho et al. 2010). This complex geology would bring populations together more readily than simply during storms or other dispersal events, and, in combination with other factors, would lead to a complex system of periods of more stable isolation and contact. Certainly a great deal of work remains to be undertaken before conclusive statements can be made in regard to the evolutionary history of these species and the collection of genetic data would be a nice supplement to such research.

## Supplementary Material

XML Treatment for Thyreus denolii

XML Treatment for Thyreus batelkai

XML Treatment for Thyreus schwarzi

XML Treatment for Thyreus aistleitneri

XML Treatment for Chiasmognathus batelkai
